# Association of Vitamin B12 with Pro-Inflammatory Cytokines and Biochemical Markers Related to Cardiometabolic Risk in Saudi Subjects

**DOI:** 10.3390/nu8090460

**Published:** 2016-09-06

**Authors:** Nasser M. Al-Daghri, Shakilur Rahman, Shaun Sabico, Sobhy Yakout, Kaiser Wani, Omar S. Al-Attas, Ponnusamy Saravanan, Gyanendra Tripathi, Philip G. McTernan, Majed S. Alokail

**Affiliations:** 1 Biomarkers Research Program, Biochemistry Department, College of Science, King Saud University, Riyadh 11451, Saudi Arabia; shakilhamdard@gmail.com (S.R.); eaglescout01@yahoo.com (S.S.); sobhy.yakout@gmail.com (S.Y.); wani.kaiser@gmail.com (K.W.); omrattas@ksu.edu.sa (O.S.A.-A.); g.tripathi@warwick.ac.uk (G.T.); msa85@yahoo.co.uk (M.S.A.); 2 Prince Mutaib Chair for Biomarkers of Osteoporosis, Biochemistry Department, College of Science, King Saud University, Riyadh 11451, Saudi Arabia; 3 Division of Health Sciences, Warwick Medical School, University of Warwick, Coventry CV4 7AL, UK; p.saravanan@warwick.ac.uk; 4 Division of Biomedical Sciences, Warwick Medical School, University of Warwick, UHCW Trust, Clifford Bridge Road, Walsgrave, Coventry CV2 2DX, UK; p.g.mcternan@warwick.ac.uk; 5 Department of Biomedical Sciences, University of Westminster, London W1W 6UW, UK

**Keywords:** vitamin B12, resistin, TNF-α, cardiometabolic diseases

## Abstract

Background: This study aimed to examine the relationship between changes in systemic vitamin B12 concentrations with pro-inflammatory cytokines, anthropometric factors and biochemical markers of cardiometabolic risk in a Saudi population. Methods: A total of 364 subjects (224 children, age: 12.99 ± 2.73 (mean ± SD) years; BMI: 20.07 ± 4.92 kg/m^2^ and 140 adults, age: 41.87 ± 8.82 years; BMI: 31.65 ± 5.77 kg/m^2^) were studied. Fasting blood, anthropometric and biochemical data were collected. Serum cytokines were quantified using multiplex assay kits and B12 concentrations were measured using immunoassay analyzer. Results: Vitamin B12 was negatively associated with TNF-α (r = −0.14, *p* < 0.05), insulin (r = −0.230, *p* < 0.01) and HOMA-IR (r = −0.252, *p* < 0.01) in all subjects. In children, vitamin B12 was negatively associated with serum resistin (r = −0.160, *p* < 0.01), insulin (r = −0.248, *p* < 0.01), HOMA-IR (r = −0.261, *p* < 0.01). In adults, vitamin B12 was negatively associated with TNF-α (r = −0.242, *p* < 0.01) while positively associated with resistin (r = 0.248, *p* < 0.01). Serum resistin was the most significant predictor for circulating vitamin B12 in all subjects (r^2^ = −0.17, *p* < 0.05) and in children (r^2^ = −0.167, *p* < 0.01) while HDL-cholesterol was the predictor of B12 in adults (r^2^ = −0.78, *p* < 0.05). Conclusions: Serum vitamin B12 concentrations were associated with pro-inflammatory cytokines and biochemical markers of cardiometabolic risks in adults. Maintaining adequate vitamin B12 concentrations may lower inflammation-induced cardiometabolic risk in the Saudi adult population.

## 1. Introduction

Vitamin B12 is an essential micronutrient required for optimal hemopoetic, neurologic and cardiometabolic function [1,2]. Human beings are dependent upon bacterial and protozoal sources to maintain adequate serum concentrations [3]. Vitamin B12 absorption is a complex process and any dysfunction can result in its deficiency, despite adequate dietary intake [4]. Vitamin B12 deficiency affects two principal enzymatic pathways, i.e., methylation process of homocysteine to methionine and the conversion of methylmalonyl coenzyme A (CoA) to succinyl-CoA [2,5], which lead to accumulation of methylmalonic acid (MMA) and/or homocysteine (Hcy) [2,5]. Excessive accumulation of homocysteine in blood (hyperhomocysteinemia) is associated with an increased risk of cardiovascular disease due to its cellular and vasculo-toxic effects [1,5]; whilst, increase in circualting methylmalonic acid (MMA) level is associated with defective fatty acid synthesis of neuronal membranes [3,5].

Current evidence demonstrated that vitamin B12 deficiency is linked to various atherogenic processes, mainly, but not exclusively, through B12 deficiency-induced hyperhomosyteinemia [6]. Hyperhomocysteinemia is a possible independent risk factor for cardiovascular disease, mechanisms of which include reduction of nitric oxide thereby promoting endothelial dysfunction [7,8]. On the other hand, cytokines, such as tumor necrosis factor (TNF-α) and resistin, play an important role in cardiovascular risk and insulin resistance [9,10].

Unfortunately, there is limited information available as to whether circulating vitamin B12 concentrations affect the cardiometabolic status of the Arab population. In fact, while it has been previously observed that vitamin B12 is inversely associated with homocysteine in the general Saudi population [11], homocysteine itself is not a risk factor for endothelial function, at least amongst Saudi Arabs [12]. Therefore, in this study we aimed to examine the associations between serum vitamin B12 concentrations, pro-inflammatory cytokines and biochemical markers of cardiovascular and metabolic risk in the general population of Saudi Arabia.

## 2. Materials and Methods

### 2.1. Subjects and Experimental Design

In this cross-sectional study, a total of 364 Saudi subjects (224 children aged 8–17 years and 140 adults aged 30–59 years) were recruited from four Primary Health Care Centers (PHCCs) within Riyadh, Saudi Arabia. Written informed consent was obtained prior to inclusion in the study. Inclusion criteria were apparently healthy children (N = 224) and adults (N = 140), nonsmokers, non-diabetic, non-hypertensive and without morbid obesity. Ethical approval was obtained from the Ethics Committee of the College of Science Research Center (Ethics Approval Code: 8/25/36681), King Saud University, Riyadh, Saudi Arabia.

### 2.2. Anthropometric Measurements

A generalized questionnaire was given to all participating subjects aimed at seeking demographic information, general health status and past medical history. Physical examination was carried out by a physician who ensured exclusion of subjects with chronic conditions (cardiac, kidney or liver disease, hypertension, psychiatric conditions) or use of medications known to affect body weight (e.g., steroids). Weight and height were recorded to the nearest 0.2 kg and 0.5 cm, respectively, using an appropriate international standard scale (Digital Pearson Scale, ADAM Equipment Inc., Oxford, CT, USA). Both measurements were done while subjects were standing upright and wearing light clothing. Body mass index (BMI) was calculated as weight in kg divided by height in squared meters (kg/m^2^). Additionally, waist and hip circumferences were measured using a standardized measuring tape in centimeters (cm) and the waist-to-hip ratio (WHR) was estimated. Blood pressure was measured using an appropriate mercury sphygmomanometer, measured twice with 5-min intervals and the mean of the two readings was recorded.

### 2.3. Blood Collection and Biochemical Measurements

Fasting blood samples were collected and stored at −20 °C. Serum insulin, leptin, adiponectin, TNF-α and resistin were quantified using multiplex assay kits that utilize fluorescent microbead technology, allowing simultaneous quantification of several target proteins within a single serum sample of 50–100 µL. These included pre-mixed and fully customized panels that utilized the Luminex’s xMAP Technology platform (Luminexcorp, TX, USA). For parameters measured using the multiplex assay, the intra-assay variation was 1.4%–7.9% and inter-assay variation of <21%. Minimum detectable concentrations (MDC) were as follows: insulin, 50.9 pg/mL; leptin, 85.4 pg/mL; adiponectin, 145.4 pg/mL; resistin, 6.7 pg/mL, and TNF-α, 0.14 pg/mL.

Vitamin B12 was measured using Roche Elecsys immunoassay analyzer, Cobas e 411 (Roche Diagnsotics GmbH, Mannheim, Germany) that employs the test principle of competitive electrochemiluminiscence immunoassay. The assay has a linearity range of 30–2000 pg/mL. The assay involves binding of vitamin B12 in the sample to the ruthenium labeled antibody. Streptavidin coated microparticles (solid phase), binds the biotin labeled vitamin B12 and ruthenium labeled antibody. A further wash period leaves the solid phase magnetic microparticles bound with vitamin B12-biotin and ruthenium labeled antibody, which generates chemiluminiscent emission signal that is inversely proportional to the concentration of vitamin B12 present in the patient sample. The inter- and intra-assay coefficients of variation (CV) for Vitamin B12 were 3.0% and 3.7%, respectively.

Fasting serum glucose concentrations and complete lipid profile (triglycerides, total cholesterol, high density lipoprotein (HDL)-cholesterol were determined using a biochemical analyzer) (Konelab, Espoo, Finland). This biochemical analyzer was calibrated routinely prior to the analysis of all serum samples using quality control samples provided by the manufacturer (Thermo-Fisher Scientific, Espoo, Finland). Low density lipoprotein (LDL)-cholesterol was calculated using the Friedman formula.

### 2.4. Statistical Analyses

Statistical analyses were carried out using the SPSS software version 16.0 (SPSS Inc., Chicago, IL, USA). Data were expressed as mean ± standard deviation. Kolmogorov-Smirnov statistics was performed to test continuous variables for normality. All non-Gaussian parameters were logarithmically or square-root transformed to normalize data. Independent Student T-test was used to compare means between groups of normally distributed data. Pearson correlations between serum vitamin B12 and the rest of the variables were determined. Stepwise linear regression models were performed using vitamin B12 as dependent variable and age, gender, BMI, systolic and diastolic blood pressure, waist and hip circumference, glucose, insulin, HDL, LDL, triglycerides, total cholesterol, leptin, adiponectin, TNF-α, resistin, HOMA-IR as independent variables to determine significant predictors. In all statistical tests, *p*-values < 0.05 were considered significant.

## 3. Results

A total of 364 Saudi subjects (224 children and 140 adults) satisfied the inclusion criteria and were included in the analysis. All the anthropometric and laboratory characteristics of the study participants are presented in Table 1.

Correlations between vitamin B12 and clinical parameters measured are presented in Table 2. In children, vitamin B12 level was negatively associated with age (r = −0.19, *p* < 0.01), hip circumference (r = −0.20, *p* < 0.01), systolic BP (r = −0.21, *p* < 0.01), insulin (r = −0.24, *p* < 0.01), HOMA-IR (r = −0.26, *p* < 0.01) and resistin (r = −0.16, *p* < 0.01). In adults, vitamin B12 was positively associated with serum resistin (r = 0.24, *p* < 0.01), whereas it was negatively associated with TNF-α (r = −0.24, *p* < 0.01). Mean serum concentrations of vitamin B12 were plotted against quartiles of HOMA-IR (supplementary materials Figure S1) and resistin (supplementary materials Figure S2) in children and adults.

Stepwise linear regression analysis, using vitamin B12 as the dependent variable and all the parameters as independent variables, is presented in Table 3. In adults, HDL-cholesterol was the significant predictor of vitamin B12 (r^2^ = 0.78, *p* = 0.044) whilst, serum resistin (r^2^ = −0.16, *p* = 0.004), gender (r^2^ = −0.15, *p* = 0.001) and age (r^2^ = −0.22, *p* = 0.02) were independent predictors of vitamin B12 level in children.

## 4. Discussion

The present study represents the first in a Saudi population and highlights a relationship between circulating vitamin B12 concentrations, cytokines and biomarkers associated with cardiometabolic risk. For this study we recruited a total of 364 subjects (224 children, and 140 adults). Fasting blood, anthropometric and biochemical data were collected. Serum cytokines were quantified using multiplex assay kits and B12 concentrations were measured using immunoassay analyzer. Our observations are of considerable clinical importance, as any irregularity in the metabolism of vitamin B12 is known to be associated with a wide spectrum of hematologic, metabolic and cardiovascular disorders and early intervention appears key [1,4,5,9].

TNF-α is a hallmark of inflammation [13] and several lines of evidence suggest the association of vitamin B12 deficiency with an increased incidence of inflammation and associated metabolic complications [3,13]. In the current study we observed a significant inverse correlation between serum vitamin B12 and TNF-α level in adults. However, in children, there was no such correlation. Elevated systemic TNF-α level is observed to be associated with depletion of primary antioxidants that cells use to protect themselves against free radicals’ damaging effects [14]. Depletion of antioxidants further induces pro-inflammatory cytokines and other inflammatory by-products like prostaglandins and leukotrienes, known risk factors for metabolic and cardiovascular diseases [14,15]. Earlier studies suggest that consistent chronic inflammation may lead to insulin resistance, type 2 diabetes mellitus (T2DM) and cardiovascular disease (CVD) [10]. Deficiency of vitamin B12 causes hyperhomocysteinemia (HHcy) [5,16], and elevated homocysteine is known to lead to an inflammatory state, a risk factor for coronary heart disease [16] and insulin resistance [17,18]. In mice, HHcy promotes insulin resistance by directly inducing the expression and secretion of TNF-α and resistin [19]. TNF-α then activates c-Jun N-terminal kinase (JNK), which, in turn, inhibits Akt activity and leads to insulin resistance [20,21].

The study also observed an inverse association between vitamin B12, HOMA-IR and insulin levels in children, indicating that deficiency of vitamin B12 at an early age may cause insulin resistance. Consistent with our observation, a similar association between vitamin B12 and HOMA-IR was observed by Mahalle et al. [1] and Kaya et al. [22], in Indian subjects with coronary artery disease and in women with polycystic ovary syndrome, respectively. Furthermore, Stewart et al. found in Nepalese subjects that low maternal vitamin B12 status was associated with insulin resistance in offspring [23]. Contrary to these, a cross-sectional study conducted in 135 Asian-Indian women found no correlation between serum vitamin B12 and HOMA-IR [24].

Resistin, originally described as an adipocyte-specific hormone, has been suggested to be an important link between obesity, insulin resistance and diabetes [25,26]. It was observed that serum concentrations of resistin are significantly increased in obese and diabetic patients [27,28]. Resistin has also been linked to the development of atherosclerosis and CVD [23,29]. In our study, a significant inverse association between vitamin B12 concentrations and resistin was observed in children, whereas, in adults, it was positively associated. Stepwise linear regression analysis revealed that resistin was the most significant predictor of vitamin B12 in children. In another study, 220 patients with acute coronary syndrome (ACS) had significantly higher serum resistin concentrations than patients classified as normal [30]. However, research on resistin has also been controversial in understanding their pathological relevance and its association with T2DM and cardiovascular diseases [31,32]. Hence, conclusive data await new strong evidence to elucidate the role of resistin in various disease processes. Nevertheless, resistin has primarily been shown to be relevant to inflammation-related diseases like atherosclerosis and arthritis [33] and may have a role in the regulation of pro-inflammatory cytokines’ (IL-6 and TNF-α) expression in human peripheral blood mononuclear cells (PBMC) via NF-κB pathway [33,34]. In yet another study conducted by Stejskal et al., it was observed that persons with clinical signs of severe inflammation showed significantly higher concentrations of resistin than healthy individuals [35]. Since resistin and TNF-α are pro-inflammatory in action, and inflammation has a central role in cardiovascular diseases and insulin resistance, deficiency of vitamin B12 may, therefore, have importance in the development of cardiometabolic diseases in Saudi population.

The limitations of this study include its cross-sectional nature and, thus, we cannot determine causality. Furthermore, information on diet is lacking and would have provided added value as to whether adequate B12 concentrations lead to better cardiometabolic parameters independent of a healthy diet. Lastly, the findings may not be a true representation of the general healthy population, since women were over represented in the current study, and whether the associations are true for those with chronic diseases also cannot be answered in the study’s present scope. Despite these limitations, this is the first study particularly from the Arab region, which reflects direct link between vitamin B12 with adipocytokines and biochemical markers of cardiometabolic risks. The present findings warrant further investigation addressing how interventional therapies with vitamin B12 affect cardiovascular and metabolic diseases.

## 5. Conclusions

In conclusion, our study suggests weak but significant associations between circulating vitamin B12 concentrations with serum insulin and HOMA-IR in children, and inflammatory cytokines such as TNF-α and resistin in adults. Our findings have significant clinical importance, since these biochemical markers and cytokines are independent risk factors for cardiovascular and metabolic diseases in adults. Thus, determination of vitamin B12 status and treatment in cases of deficiency could be a clinically useful strategy in the prevention of cardiometabolic diseases.

## Figures and Tables

**Table 1:** Clinical characteristics, glycemic, lipid and metabolic profiles of subjects.

Parameters	Children	Adults
Clinical Characteristics		
N	224	140
M/F (%)	122/102 (54.5/45.5)	37/103 (26.4/73.6)
Age (years)	12.99 ± 2.73	41.87 ± 8.82
Body Mass Index (kg/m^2^)	20.07 ± 4.92	31.65 ± 5.77
Waist circumference (cm)	66.37 ± 15.50	88.51 ± 15.65
Hip circumference (cm)	82.06 ± 15.59	99.01 ± 15.77
Systolic Blood Pressure	105.54 ± 10.21	114.25 ± 13.53
Diastolic Blood Pressure	68.85 ± 7.12	75.73 ± 8.81
Glycemic Profile		
Glucose (mmol/L)	5.11 ± 0.61	5.37 ± 0.81
Insulin (IU/mL) #	6.5 (4.1–10.8)	9.3 (7.1–13.2)
HOMA-IR #	1.46 (0.88–2.41)	2.37 (1.50–3.22)
Lipid Profile		
Triglycerides (mmol/L) #	1.06 ± 0.55	1.54 ± 0.71
Total Cholesterol (mmol/L)	4.23 ± 0.77	4.65 ± 0.98
HDL-Cholesterol (mmol/L)	1.02 ± 0.34	0.87 ± 0.33
LDL-Cholesterol (mmol/L)	2.72 ± 0.69	3.07 ± 0.77
Metabolic Profile		
Leptin (ng/mL) #	9.10 (1.94–27.62)	15.73 (7.52–25.67)
Adiponectin (mg/mL) #	20.22 (12.66–28.42)	17.37 (5.19–431)
Resistin (ng/mL) #	15.10 (9.97–20.88)	5.01 (2.89–14.47)
TNF-α (pg/mL) #	7.51 (4.91–9.79)	15.34 (5.08–74.02)
Vitamin B12 (Pg/mL) #	421 (290.6–530.6)	371.25 (288.6–496.3)

Note: Data presented as N (%) for frequencies; mean ± standard deviation for normal continuous variables; # denotes continuous variables with non-Gaussian distribution and presented as median (25th–75th Percentile).

**Table 2:** Associations between vitamin B12 and clinical, glycemic, lipid and metabolic profiles.

Parameters	Coefficients of Correlation (r)	
	Children (N = 224)	Adults (N = 140)
Age (years)	−0.192 **	0.022
Body Mass Index (kg/m^2^)	−0.132	0.006
Waist circumference (cm)	−0.105	0.089
Hip circumference (cm)	−0.207 **	0.050
Systolic Blood Pressure (mmHg)	−0.211 **	0.116
Diastolic Blood Pressure (mmHg)	−0.119	−0.029
Glucose (mmol/L)	−0.102	−0.071
Insulin (IU/mL) #	−0.248 **	−0.118
HOMA-IR	−0.261 **	−0.156
Triglyceride (mmol/L) #	−0.104	0.041
Cholesterol (mmol/L)	0.010	0.177
HDL-Cholesterol (mmol/L)	0.139 *	0.030
LDL-Cholesterol (mmol/L)	−0.046	0.117
Leptin (ng/mL) #	−0.130	0.060
Adiponectin (mg/mL) #	0.054	0.036
Resistin (ng/mL) #	−0.160 **	0.248 **
TNF-α (pg/mL) #	−0.062	−0.242 **

Note: Data presented as coefficient (r); * denotes significance at 0.05 level; ** denotes significance at 0.01 level; # log transformed values.

**Table 3:** Stepwise linear regression analysis using vitamin B12 as a dependent variable.

	Predictor	Unstandardized β	SE	*p*-Value
Adults	HDL-cholesterol	0.781	0.169	0.044
Children	Gender	−0.155	0.036	0.001
	Age	−0.022	0.007	0.02
	Resistin	−0.167	0.069	0.004

Note: Independent variables entered: age, gender, BMI, systolic and diastolic blood pressure, waist and hip circumference, glucose, insulin, HDL-cholesterol, LDL-cholesterol, triglycerides, total cholesterol, leptin, adiponectin, TNF-α, resistin, HOMA-IR. Significant at *p* < 0.05.

## Supplementary Materials

The following are available online at http://www.mdpi.com/2072-6643/8/9/460/s1, Figure S1: Mean vitamin B12 concentrations according to quartiles of HOMA-IR in children and adults, Figure S2: Mean vitamin B12 concentrations according to quartiles of resistin in children and adults.
