# Supplementary material for: Association of Vitamin B12 with Pro-Inflammatory Cytokines and Biochemical Markers Related to Cardiometabolic Risk in Saudi Subjects
Source: Nutrients. 2016 Sep 6;8(9):460. doi: 10.3390/nu8090460 (PMC5037505; doi:10.3390/nu8090460)
Supplement: Supplementary file 1 [file nutrients-08-00460-s001.docx]

Supplementary Materials: Association of Vitamin B12 with Pro-inflammatory Cytokines and Biochemical Markers Related to Cardiometabolic Risk in Saudi Subjects

Nasser M. Al-Daghri, Shakilur Rahman, Shaun Sabico, Sobhy Yakout, Kaiser Wani, Omar S. Al-Attas, Ponnusamy Saravanan[, Gyanendra Tripathi,](http://diabetes.diabetesjournals.org/search?author1=Philip+G.+McTernan&sortspec=date&submit=Submit)[Philip G. McTernan](http://diabetes.diabetesjournals.org/search?author1=Philip+G.+McTernan&sortspec=date&submit=Submit) and Majed S. Alokail

**Figure S1.** Mean vitamin B12 concentrations according to quartiles of HOMA-IR in children and adults.

**Figure S2.** Mean vitamin B12 concentrations according to quartiles of resistin in children and adults.
